# An evolutionary molecular adaptation of an unusual stefin from the liver fluke *Fasciola hepatica* redefines the cystatin superfamily

**DOI:** 10.1016/j.jbc.2023.102970

**Published:** 2023-02-01

**Authors:** Michal Buša, Zuzana Matoušková, Pavla Bartošová-Sojková, Petr Pachl, Pavlína Řezáčová, Ramon Marc Eichenberger, Peter Deplazes, Martin Horn, Saša Štefanić, Michael Mareš

**Affiliations:** 1 Institute of Organic Chemistry and Biochemistry, Czech Academy of Sciences, Prague, Czechia; 2 Department of Biochemistry, Faculty of Science, Charles University, Prague, Czechia; 3 Institute of Parasitology, Biology Centre, Czech Academy of Sciences, Budweis, Czechia; 4 Institute of Parasitology, University of Zurich, Zurich, Switzerland

**Keywords:** cystatin, stefin, protease inhibitor, cysteine cathepsin, protein structure, protein evolution, helminth parasite, NEJ, newly excysted juveniles, E/S, excretory/secretory, CyLS, cystatin-like stefins, FhCyLS-2, *Fasciola hepatica* cystatin-like stefin 2, LC-MS/MS, liquid chromatography–tandem mass spectrometry, qRT-PCR, quantitative RT-PCR

## Abstract

Fasciolosis is a worldwide parasitic disease of ruminants and an emerging human disease caused by the liver fluke *Fasciola hepatica*. The cystatin superfamily of cysteine protease inhibitors is composed of distinct families of intracellular stefins and secreted true cystatins. FhCyLS-2 from *F. hepatica* is an unusual member of the superfamily, where our sequence and 3D structure analyses in this study revealed that it combines characteristics of both families. The protein architecture demonstrates its relationship to stefins, but FhCyLS-2 also contains the secretion signal peptide and disulfide bridges typical of true cystatins. The secretion status was confirmed by detecting the presence of FhCyLS-2 in excretory/secretory products, supported by immunolocalization. Our high-resolution crystal structure of FhCyLS-2 showed a distinct disulfide bridging pattern and functional reactive center. We determined that FhCyLS-2 is a broad specificity inhibitor of cysteine cathepsins from both the host and *F. hepatica*, suggesting a dual role in the regulation of exogenous and endogenous proteolysis. Based on phylogenetic analysis that identified several FhCyLS-2 homologues in liver/intestinal foodborne flukes, we propose a new group within the cystatin superfamily called cystatin-like stefins.

The common liver fluke, *Fasciola hepatica*, is a globally spread parasitic flatworm of the class Trematoda that infects mainly sheep and cattle, causing the disease fasciolosis. It negatively affects livestock production with estimated worldwide losses of €2.5 billion annually (1). Fasciolosis is an important zoonosis with 2.4 million people infected, and 150 million in over seventy countries at risk, and is classified by the World Health Organization as a neglected tropical disease. The control of fasciolosis relies on a single drug, triclabendazole, and reports of resistant strains of *F. hepatica* are on the rise (2).

The superfamily of cystatins (Merops database ID: I25) is ubiquitous in a wide range of organisms, including animals, plants, fungi, and protista. The superfamily members are reversible, tight-binding inhibitors of cysteine proteinases; they possess a reactive center against papain-family proteases, and some members contain an additional reactive center against legumains (3, 4, 5). Based on their sequence and spatial structure, cystatins can be divided into three families: the type 1 family of stefins (Merops ID: I25A) with intracellular single-domain proteins devoid of the signal sequence and disulfide bridges, the type 2 family of true cystatins (Merops ID: I25B) with secreted single-domain proteins containing two disulfide bridges, and the type 3 family of kininogens (Merops ID: I25C) with multidomain proteins composed of I25B repeats (5, 6). The most characterized are mammalian cystatins, which were shown to regulate a broad range of physiological and pathological processes, including protein catabolism, hormone processing, bone resorption, antigen processing, inflammation, and tumor metastasis (for review, see, *e.g.*, (5, 7, 8, 9)).

In parasites, cystatins are not only essential in the regulation of physiological processes during parasite development but also represent important pathogenicity factors. Various reports indicated that parasite cystatins have evolved exceptional immunomodulatory properties. Most of the examples studied originated from ticks and helminths. In ticks, it was shown that gut-associated cystatins regulate endogenous digestive proteases, while salivary cystatins that are injected into the host can inhibit the secretion of proinflammatory cytokines, reduce T-cell proliferation, or disrupt dendritic cell maturation and differentiation (10, 11). Helminth cystatins were demonstrated to block antigen processing and presentation, interfere with the processing of pattern recognition receptors in innate immunity, modulate production of cytokines and nitric oxide, and suppress T-cell proliferation (12, 13, 14, 15, 16).

*F. hepatica* cysteine proteases from the papain family (cysteine cathepsins) represent about 80% of secreted proteolytic activity of the adult parasite and belong to cathepsins L and B. They are involved in invasion, migration through the host body, feeding, and immune evasion and are regulated by several mechanisms, including activation processing, compartmentalization, and interaction with protease inhibitors (17, 18, 19, 20).

Previous studies identified several inhibitors of cysteine cathepsins in *F. hepatica*, including stefins, a multidomain cystatin, and a Kunitz protein (21, 22, 23), which were suggested as candidate targets for a fasciolosis vaccine (24, 25). A similar distribution of inhibitors has been reported for a closely related species, *Fasciola gigantica* (26, 27). No *Fasciola-*derived member of the cystatin superfamily has yet been structurally characterized, and in general, our knowledge of helminth cystatins at the structure-function level is very limited (28, 29).

In this work, we investigated the cysteine cathepsin inhibitor FhCyLS-2 from *F. hepatica*, belonging to the cystatin superfamily, and analyzed its crystal structure, inhibitory properties, localization, and phylogeny. Based on distinct molecular features of FhCyLS-2 and its homologues, we defined a new evolutionary group called cystatin-like stefins (CyL-stefins, CyLS) within the cystatin superfamily, with FhCyLS-2 as a prototype member characterized at the structural and functional levels.

## Results

### FhCyLS-2 sequence combines characteristics of stefins and true cystatins

**Figure 1:**
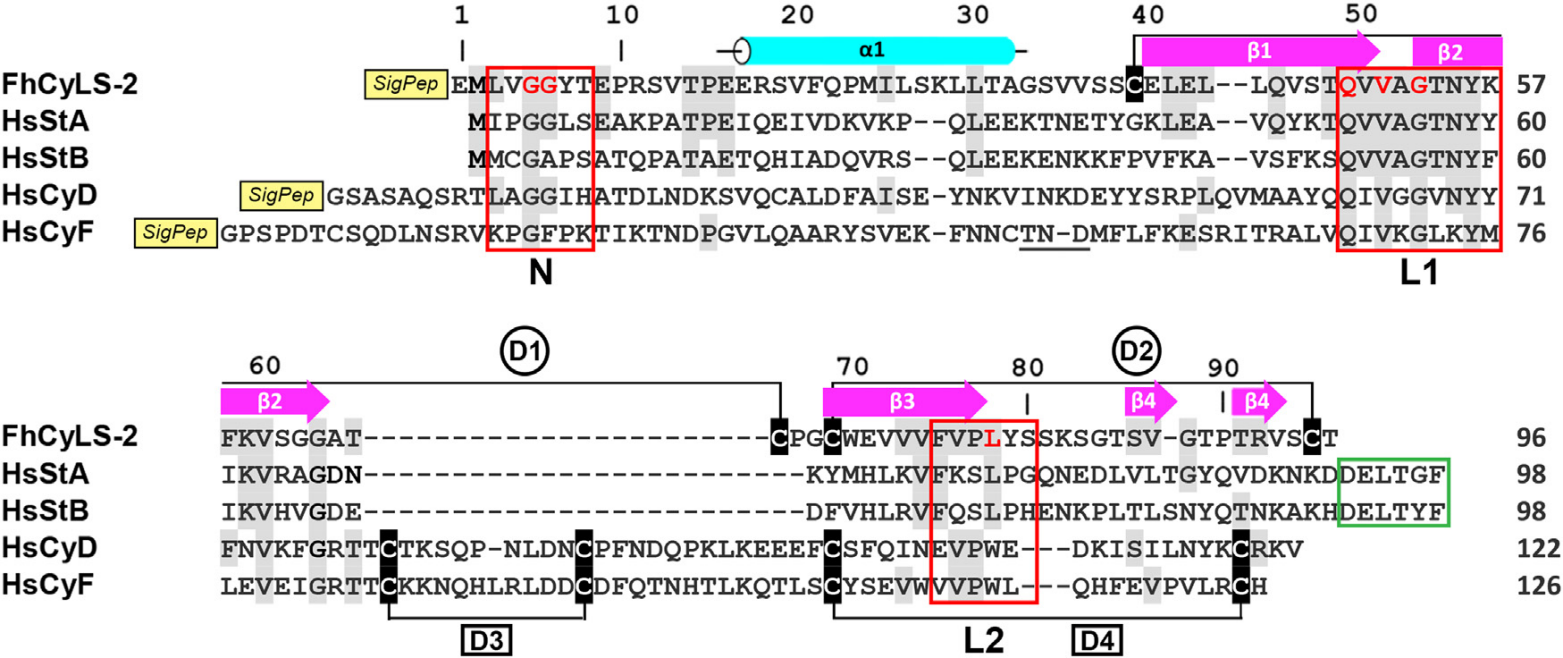
**Structure-based sequence alignment of FhCyLS-2 with representative members of cystatin superfamily.** FhCyLS-2 is compared with representative human members of the type 1 family, stefins A and B (HsStA and HsStB, respectively), and the type 2 family, cystatins D and F (HsCyD and HsCyF, respectively). Residues identical with those of FhCyLS-2 are shaded *gray*; cysteine residues are shaded *black*. The disulfide bridges are indicated by the connecting *black lines* and labeled (*circled* D1 and D2 for FhCyLS-2, *boxed* D3 and D4 for cystatins D/F). The secondary structure elements of FhCyLS-2 are depicted in *cyan* for α-helix and *magenta* for β-strands and labeled as in Fig. 5*A*. Three segments forming the reactive center for inhibition of cysteine cathepsins are *boxed in red* and labeled as in Fig. 5*A* (the region size was selected based on the predominant binding residues in the available complex structures); the critical consensus positions are highlighted in *red* for FhCyLS-2. The conserved C-terminal segment of classical stefins is *boxed in green*. Mature protein sequences without signal peptides (indicated by a SigPep box) were used in the alignment; residue numbering on the top line is according to FhCyLS-2. GenBank accessions: FhCyLS-2, AAV68752; HsStA, AAH10379; HsStB, AAH10532; HsCyD, AAH62678; HsCyF, CAG46658.

The full-length cDNA available in GenBank under accession no. AY647146.1 contains an open reading frame coding for a 116-amino-acid protein (GenBank accession no. AAV68752), which we designated here as *F. hepatica* cystatin-like stefin 2 (FhCyLS-2). In Figures 1 and S1 we compared the sequence of FhCyLS-2 with that of other members of the cystatin superfamily, including representative vertebrate members of the type 1 family (stefins) and type 2 family (true cystatins) (Fig. 1) and selected invertebrate members of both families from various blood feeding parasites (Fig. S1). The sequence alignments revealed several important features in the FhCyLS-2 sequence, which allowed us to assess the relationship between FhCyLS-2 and both families.

Our results showed that FhCyLS-2 and stefins did not contain a large insert in the central part of the molecule, which is seen in true cystatins (between strands β2 and β3, Figs. 1 and S1). On the other hand, FhCyLS-2 lacked a C-terminal sequence motif Asp/Glu-Xxx-Leu-Xxx-Tyr/His-Phe (30) typical for stefins (Figs. 1 and S1). The interaction of all cystatin superfamily members with cysteine cathepsins is mediated by three regions, including the N-terminal segment and two hairpin loops L1 and L2 (3, 31). In FhCyLS-2, the mature N terminus carried the Gly-Gly motif and the L1 loop bore the Gln-Xxx-Val-Xxx-Gly motif; both motifs are conserved among stefins and true cystatins (Figs. 1 and S1). The L2 loop was characterized in stefins and FhCyLS-2 by the presence of a conserved central Leu residue, in contrast to a Trp residue in true cystatins (Fig. 1 and S1). To summarize, FhCyLS-2 possessed the sequence insertion/deletion pattern and signature of a functionally competent reactive center for inhibition that were related to those present in stefins.

Analysis of the FhCyLS-2 sequence identified a signal peptide composed of 20 N-terminal residues with a predicted cleavage site between residues Gly and Glu1 (the first residue in the mature protein) (Fig. S1). The signal peptide is a hallmark of true cystatins, which are secreted proteins, and distinguish them from stefins localized intracellularly due to the lack of the signal peptide (6, 32).

The FhCyLS-2 sequence also contained four cysteine residues, and we investigated their redox status (*i.e.*, free or disulfide bonded). Disulfide bridges are a typical feature of true cystatins; however, they are absent in stefins, which are usually devoid of cysteine residues or contain unpaired cysteines with a free thiol (6, 32). We prepared recombinant FhCyLS-2 and performed liquid chromatography–tandem mass spectrometry (LC-MS/MS) peptide mapping. Analysis of disulfide-linked peptide clusters revealed that the cysteine residues formed two disulfide bridges with connectivity Cys39-Cys66 and Cys69-Cys95 (Table S1). They are labeled as D1 and D2, respectively, in the FhCyLS-2 sequence in Figure 1. Thus, FhCyLS-2 resembles true cystatins containing two disulfides; a comparison of their disulfide patterns is discussed in the section on the 3D structure of FhCyLS-2.

In conclusion, we demonstrated that FhCyLS-2 was a close homologue of the stefin family due to sequence similarity. However, at the same time, FhCyLS-2 also displayed two hallmarks of the family of true cystatins, namely, two disulfides and the N-terminal signal sequence for secretion. Furthermore, in the sequence databases we found a set of stefin sequences of trematode origin, which were highly homologous to FhCyLS-2 and also contained the signal peptide as well as putative disulfides (Fig. S1). Based on this finding, we propose a new group of the cystatin superfamily named cystatin-like stefins (CyL-stefins) with FhCyLS-2 as a first member. In the following chapters, we will analyze FhCyLS-2 in detail; regarding the signal sequence function, disulfide-bridged 3D structure, and phylogeny of CyL-stefins.

### *F. hepatica* developmental stages express and secrete FhCyLS-2

**Figure 2:**
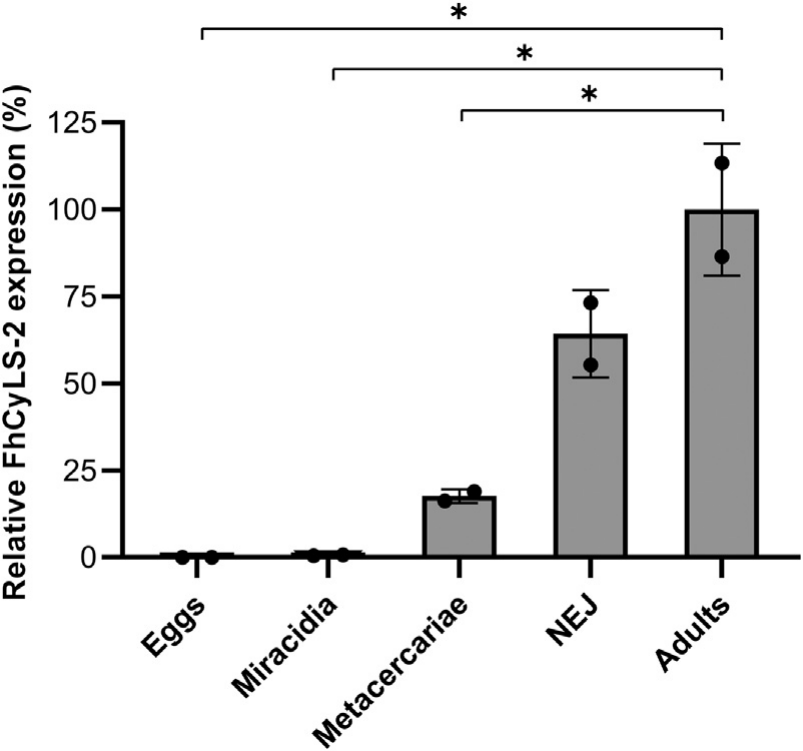
**Transcriptional profiling of FhCyLS-2 in the developmental stages of *F. hepatica*.** The expression of FhCyLS-2 was evaluated by qRT-PCR. mRNA transcriptional levels are presented as percentage of expression relative to that in adults. The mean values ± SD for pooled biological samples are given; statistical significance compares adults with other stages (∗*p* < 0.05). NEJ, newly excysted juveniles.

The mRNA transcript levels for FhCyLS-2 were evaluated in eggs, miracidia, metacercariae, newly excysted juveniles (NEJs), and adults by quantitative RT-PCR (qRT-PCR) (Fig. 2); intramolluscan stages and cercariae were not investigated. The highest expression of FhCyLS-2 was recorded in adults followed by NEJ and metacercariae. Expression in miracidia was below 1% of the FhCyLS-2 level in adults, and no expression was detected in eggs. The transcript level increased gradually with development from miracidia to adult.

The excretory/secretory (E/S) products collected from adult *F. hepatica* were subjected to proteomic analysis to directly demonstrate the presence of FhCyLS-2. The LC-MS/MS strategy was based on enzymatic digestion of a complex protein mixture and MS/MS peptide sequencing. This analysis provided 22% peptide coverage of the FhCyLS-2 sequence, allowing us to conclude that FhCyLS-2 was secreted into the E/S products of *F. hepatica*. Furthermore, using this approach we determined the N terminus of native FhCyLS-2 (Table S1) and demonstrated that mature FhCyLS-2 was produced by cleavage of the signal peptide at the Gly(-1)-Glu1 bond (Fig. S1).

### FhCyLS-2 is localized in the worm gut, reproductive system, and tegument surface

**Figure 3:**
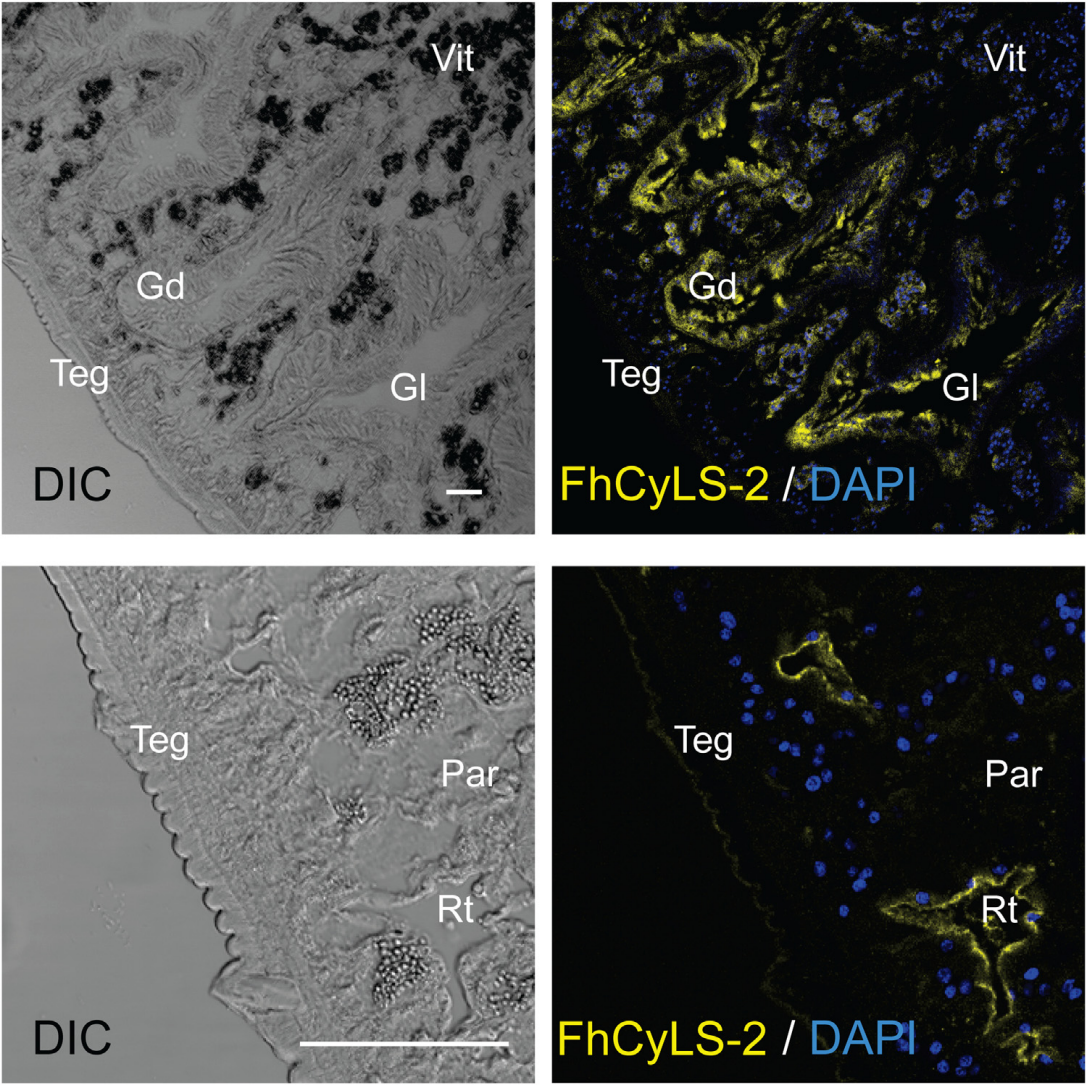
**FhCyLS-2 immunolocalization in sections of an adult *F. hepatica*.** Semithin tissue sections of adult worms were probed with an anti-FhCyLS-2 antiserum followed by reaction with an anti-mouse IgG Alexa 633–labeled secondary antibody (*yellow*). DAPI was used to label nuclear DNA (*blue*). The *left column* shows differential interference contrast (DIC), the *right column* fluorescent channels. *Upper panels*: FhCyLS-2 localized to the gastroderm of the adult worm (Gd, gastrodermal cells; Gl, gut lumen; Teg, tegument; Vit, vitelline cells). *Lower panels*: Higher magnification revealed a faint signal on the tegument surface and an intense signal lining the ducts of presumably reproductive organs (Rt, reproductive tract; Par, parenchymal tissue; Teg, tegument). The scale bars represent 100 μm; negative control staining is shown in Fig. S2.

Indirect immunofluorescence microscopy on semithin sections using polyclonal antibodies raised against recombinant FhCyLS-2 demonstrated that FhCyLS-2 was expressed in distinct tissues of adult worms (Fig. 3). FhCyLS-2 was observed in the intestinal epithelium and the reproductive tract of adult parasites; a much weaker signal was present on tegument surface. FhCyLS-2 was absent in parenchyma or vitelline cells. Preimmune serum was used as a negative control, and only faint background fluorescence was detected (Fig. S2).

### Preparation of recombinant FhCyLS-2

**Figure 4:**
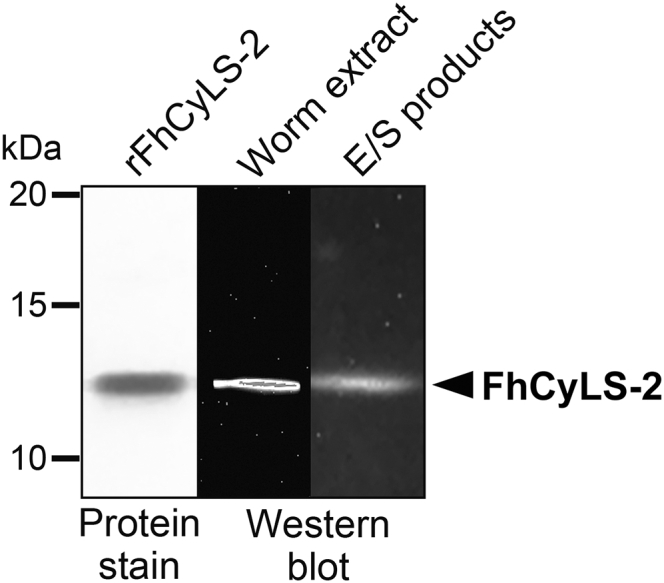
**Preparation of recombinant FhCyLS-2 and identification of native FhCyLS-2.** The recombinant FhCyLS-2 (rFhCyLS-2) expressed in *P. pastoris* was resolved by SDS-PAGE and protein-stained. Protein extract of adult *F. hepatica* and the excretory/secretory (E/S) products were visualized by Western blotting using mouse polyclonal antibodies raised against rFhCyLS-2. The position of FhCyLS-2 is indicated.

Recombinant FhCyLS-2 was prepared as a mature protein with an oligohistidine tag appended to its C terminus (predicted mass of 11,698 Da) and expressed using a *Pichia pastoris* system. The protein purified to homogeneity (see Experimental procedures) migrated on SDS-PAGE as a single band of ∼12 kDa (Fig. 4). Its identity was confirmed by LC-MS/MS analysis with complete peptide coverage. Mouse polyclonal antibodies, raised against recombinant FhCyLS-2, were used for the detection of native FhCyLS-2. In the E/S products and tissue extract of adult *F. hepatica*, the antibody recognized a band of ∼12 kDa corresponding to FhCyLS-2 (Fig. 4), which indicated that FhCyLS-2 was released into the host environment.

### FhCyLS-2 is a potent inhibitor of mammalian and *F. hepatica* cysteine cathepsins

The purified recombinant FhCyLS-2 was screened *in vitro* for its inhibitory activity against a panel of cysteine proteases; its inhibitory profile is shown in Table 1. We demonstrated that FhCyLS-2 interacts with proteases of the CA but not the CD clan from the cysteine protease class. This was tested with papain and legumain (Table 1), which are representative members of the CA and CD clans, respectively, and archetypes in cystatin research (3, 33). A set of papain-family proteases (family C1, clan CA) of mammalian origin was then screened, including cathepsins F, K, L, S, and V (endopeptidases); cathepsins B and X (a peptidyl dipeptidase/endopeptidase and carboxypeptidase, respectively); and cathepsins C and H (a dipeptidyl peptidase and aminopeptidase, respectively). These enzymes were selected to cover a wide range of endo- and exopeptidase cleavage specificities. FhCyLS-2 strongly inhibited all these cysteine cathepsins, with IC_50_ values ranging from approximately 0.9 to 8.6 nM, except for cathepsins F and C (IC_50_ of 49 and 66 nM, respectively) and cathepsin X (IC_50_ of 8.4 μM), which were inhibited with lower potency (Table 1). Furthermore, FhCyLS-2 was tested against three major *F. hepatica* cathepsin L proteases, which are important *Fasciola* virulence factors: cathepsins L1 (FhCL1) and L2 (FhCL2) expressed in immature and mature flukes, and cathepsin L3 (FhCL3) from infectious larvae (18). These recombinant cysteine cathepsins from *F. hepatica* were highly sensitive to inhibition, with IC_50_ values in the low nanomolar range (2.5–18.1 nM) (Table 1).

**Table 1:** Inhibitory effect of FhCyLS-2 on the activity of cysteine proteases

Enzyme	IC_50_ (nM)	Enzyme specificity, protease clan/family
Mammalian cathepsins		
Cathepsin L	0.92 ± 0.01	Endopeptidase, CA/C1
Cathepsin S	1.1 ± 0.1	Endopeptidase, CA/C1
Cathepsin B	2.1 ± 0.2	Peptidyl dipeptidase/endopeptidase, CA/C1
Cathepsin H	2.5 ± 0.2	Aminopeptidase, CA/C1
Cathepsin V	5.9 ± 0.3	Endopeptidase, CA/C1
Cathepsin K	8.6 ± 0.7	Endopeptidase, CA/C1
Cathepsin F	49 ± 5	Endopeptidase, CA/C1
Cathepsin C	66 ± 7	Dipeptidyl peptidase, CA/C1
Cathepsin X	8400 ± 800	Carboxypeptidase, CA/C1
*Fasciola hepatica* cathepsins		
Cathepsin L1	2.5 ± 0.5	Endopeptidase, CA/C1
Cathepsin L2	10 ± 1	Endopeptidase, CA/C1
Cathepsin L3	18 ± 1	Endopeptidase, CA/C1
Others		
Papain	1.1 ± 0.1	Endopeptidase, CA/C1
Legumain	n.i.	Endopeptidase, CD/C13


The inhibitory potency of FhCyLS-2 was determined against papain-family proteases from the CA clan and legumain from the CD clan. The IC_50_ values (means ± SEM) were determined by a kinetic activity assay using specific fluorogenic peptide substrates. The Merops database classification of the cysteine proteases tested (clan/family) and their mode of action are given. n.i. indicates no significant inhibition at 10 μM inhibitor concentration.

We also investigated the effect of FhCyLS-2 on the proteolytic activity of native cysteine cathepsins in the E/S products of adult *F. hepatica*, which were attributed mainly to FhCL1 and FhCL2 (18). Activity measured using a specific peptide substrate was almost completely inhibited by FhCyLS-2 in agreement with the data obtained for recombinant *F. hepatica* cathepsins L (Table S2). This result was further supported by evaluation of FhCyLS-2 in an assay using general protein substrates, including physiologically relevant collagen and elastin. It showed that FhCyLS-2 was capable of effectively blocking most proteolytic activity of the E/S products (Table S2).

In summary, FhCyLS-2 was identified as a broad-specificity inhibitor targeting various cysteine cathepsins, including exogenous mammalian enzymes and endogenous digestive enzymes secreted by *F. hepatica*.

### Three-dimensional structure of FhCyLS-2: a unique disulfide-bridged stefin-like architecture

The crystal structure of recombinant FhCyLS-2 was determined by single isomorphous replacement and refined using native data to 1.60 Å resolution. FhCyLS-2 crystallized in an orthorhombic space group *C*222_1_ with one molecule in the asymmetric unit and a solvent content of 48.3% (Table S3). All protein residues could be modeled into a well-defined electron density map except for the first three N-terminal residues (Glu1–Leu3) and the C-terminal oligohistidine tag (Ala97–His105). The final model of FhCyLS-2 contained 93 residues (Val4–Thr96).

**Figure 5:**
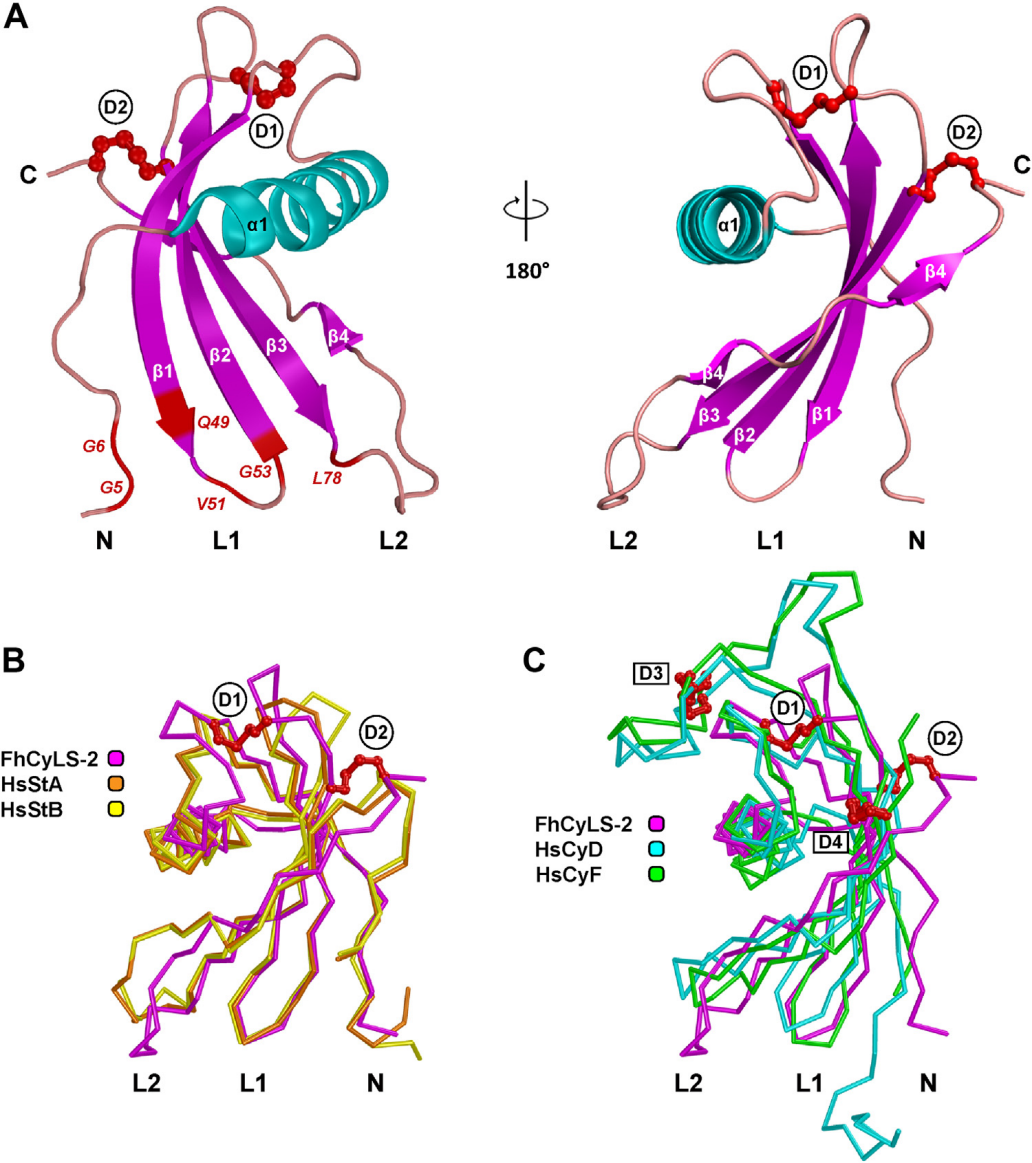
**The crystal structure of FhCyLS-2 and its comparison with cystatins from type 1 and type 2 families.***A*, the 3D structure of FhCyLS-2 (PDB code 6I1M) is shown in a cartoon representation colored by secondary structure elements (α1 helix in *cyan*, β1–β4 strands in *magenta*). The N and C termini (labelled N and C, respectively) and two disulfide bridges Cys39-Cys66 (D1) and Cys69-Cys95 (D2) (represented by *red sticks* and *balls*) are indicated. The hairpin loops L1 and L2 and the N terminus are involved in the binding of inhibitors from the cystatin superfamily to cysteine cathepsins; the predicted critical residues are highlighted and labeled in *red*. *B and C*, superposition of Cα traces of FhCyLS-2 (*magenta*) with human stefins *A* and *B* (*orange* and *yellow*, respectively) and with human cystatins *D* and *F* (*cyan* and *green*, respectively). Disulfide bridges are shown as *red sticks* and *balls* and labeled as in Figure 1. The segments N, L1, and L2 forming the reactive center against cysteine cathepsins are defined in Figure 1. The orientation of FhCyLS-2 is as in *A* (the *right-hand view*). PDB accessions: FhCyLS-2, 6I1M; stefin A (protease bound), 3KSE; stefin B (protease bound),1STF; cystatin D, 1RN7; cystatin F, 2CH9.

Figure 5*A* shows the overall structure of FhCyLS-2 formed by a four-stranded twisted antiparallel β-sheet, which were wrapped around a central α-helix. The molecule adopted a typical fold of classical stefins like the human homologues stefins A and B (Fig. 5*B*). The closest structural homologue of FhCyLS-2 was stefin B with the lowest RMSD for Cα (1.98 Å), followed by stefin A (2.14 Å RMSD) (Fig. 5*B*); lower structural similarities were found with true cystatins represented by human cystatins D and F (3.90 Å and 3.34 Å RMSD, respectively) (Fig. 5*C*).

In contrast to stefins A and B, the intact C-terminal strand β4 was split into two smaller β-strands in FhCyLS-2 (Figs. 1 and 5*A*). Importantly, FhCyLS-2 incorporated two disulfide bridges into the disulfide-free scaffold of stefins. They were located in loops at the edge of the β-sheet and indicated as D1 (Cys39-Cys66) and D2 (Cys69-Cys95) (Fig. 5*A*). However, the two-disulfide pattern of FhCyLS-2 differed from that conserved in true cystatins (their disulfides are indicated as D3 and D4 in Figs. 1*C* and 5). While the positions of the C-terminal disulfides (D2 and D4) were similar, the upstream disulfides formed an interloop bridge (D1) in FhCyLS-2 but an intraloop bridge (D3) in the cystatins D and F (Figs. 1 and 5*C*). All disulfides were located on the side of the molecules opposite to the reactive center (Fig. 5*C*).

In both stefins and true cystatins, the reactive center creates a tripartite wedge-shaped edge formed by the N-terminal trunk and two hairpin loops L1 and L2, which binds into the active site cleft of cysteine cathepsins (3, 31). In FhCyLS-2 (Figs. 1 and 5*A*), the N-terminal trunk was located around the conserved pair of glycines (Gly5, Gly6) providing conformational flexibility to this reactive segment in order to adopt an optimal conformation for target binding. The L1 loop (between β1 and β2) of FhCyLS-2 exposed the segment Gln49-Val50-Val51-Ala52-Gly53 corresponding to the critical binding motif Gln-Xaa-Val-Xaa-Gly. The L2 loop (between β3 and β4) was characterized in FhCyLS-2 and classical stefins by the presence of a conserved Leu78 residue.

In conclusion, the crystallographic analysis demonstrated that FhCyLS-2 is a structurally distinct member of the type 1 family of stefins equipped with a unique pattern of disulfide bridges and a functionally competent reactive site.

### Phylogenetic analysis of FhCyLS-2 and cystatin-like stefins

**Figure 6:**
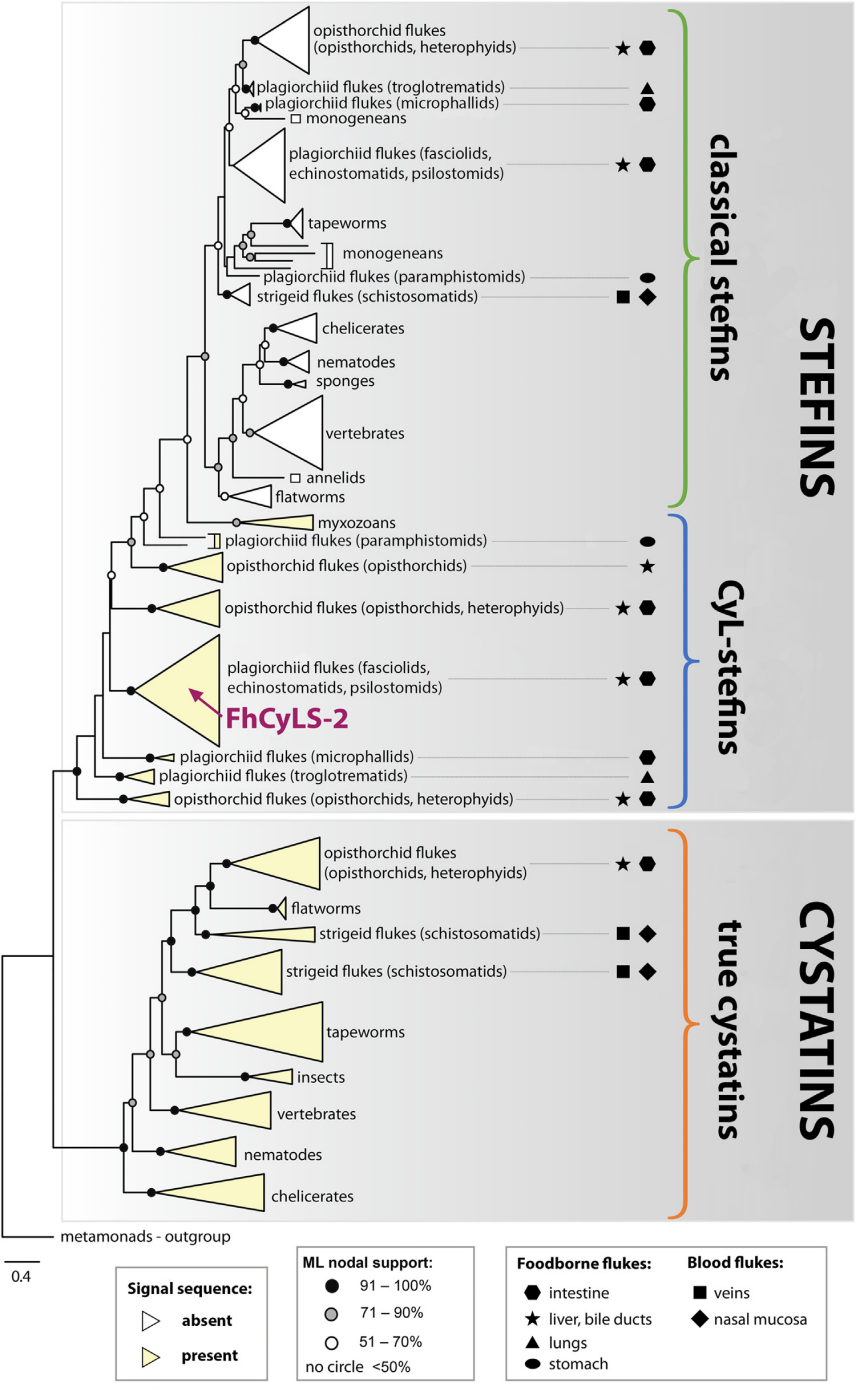
**Maximum likelihood phylogenetic tree of the cystatin superfamily.** The rooted tree of vertebrate and invertebrate members shows two clades of the type 1 family of stefins and the type 2 family of true cystatins; the former is divided into classical stefins and cystatin-like stefins (CyL-stefins). The position of *F. hepatica* FhCyLS-2 is marked. Note that sequences of CyL-stefins and true cystatins contain the signal peptide (*yellow-colored* taxa/clades). The stem group of CyL-stefins covers foodborne flukes (Trematoda) and myxozoans (Cnidaria). The organ location of mature fluke stages in the definitive host is indicated by symbols for each group. The metamonad *Giardia intestinalis* cystatin, related to an ancestral gene of the superfamily (6), was used as the outgroup. Nodal supports calculated from 1000 bootstrap replicates are depicted as dots on each node. The detailed phylogenetic tree with taxa names and input sequences are provided in Fig. S3 and File S1.

Phylogenetic analysis of FhCyLS-2 and other monomeric members of the cystatin superfamily is presented in Figure 6. It shows two main monophyletic clades of type 1 and type 2 families, corresponding to stefins and true cystatins, respectively. Within the major clades, all sequences were grouped into several taxonomy-defined subclades. Importantly, stefins were divided into a monophyletic clade of classical stefins and a stem group of cystatin-like stefins (CyL-stefins) including FhCyLS-2. Sequences of CyL-stefins were distinct from classical stefins by containing the signal sequence (as indicated in Fig. 6); this feature was also present in true cystatins. All identified CyL-stefins originated from certain taxa of parasitic Trematoda and Cnidaria, namely, the foodborne flukes (Trematoda) infecting the gastrointestinal, biliary, and respiratory tract of their definitive vertebrate hosts (fasciolids, echinostomatids, psilostomids, paramphistomids, microphalids, troglotrematids, opisthorchiids, heterophyids) and the myxozoans (Cnidaria). Interestingly, no CyL-stefins were identified in the blood flukes (schistosomatids) (Fig. 6).

*F. hepatica* FhCyLS-2 (GenBank: AY64714) firmly grouped with two paralogs from the same species (Genbank: THD26684, THD28140) and with the orthologs from other liver and intestinal foodborne flukes (Plagiorchiida: fasciolids, echinostomatids, psilostomids), forming the largest clade of CyL-stefins (Fig. 6). The only other sequence from the cystatin superfamily found in *F. hepatica* (Genbank: THD19897) belonged to the clade of classical stefins (Figs. S1 and S3). Thus, no true cystatin homologue was identified for *F. hepatica* and for other Plagiorchiida. In contrast, members of Opisthorchiida and Schistosomatida possessed homologues that clustered in the clade of true cystatins.

In conclusion, our phylogenetic analysis revealed that FhCyLS-2 is formally a member of the type 1 family, stefins. However, FhCyLS-2 and its homologues, defined as CyL-stefins from Trematoda and Myxozoa species, were evolutionarily different from classical stefins and formed a distinct stem group in the phylogenetic branch of the type 1 family. *F. hepatica* and all other species from the order Plagiorchiida encoded both CyL-stefins and classical stefins but lacked genes for true cystatins.

### FhCyLS-2 is recognized by serum from naturally and experimentally infected ruminants

**Figure 7:**
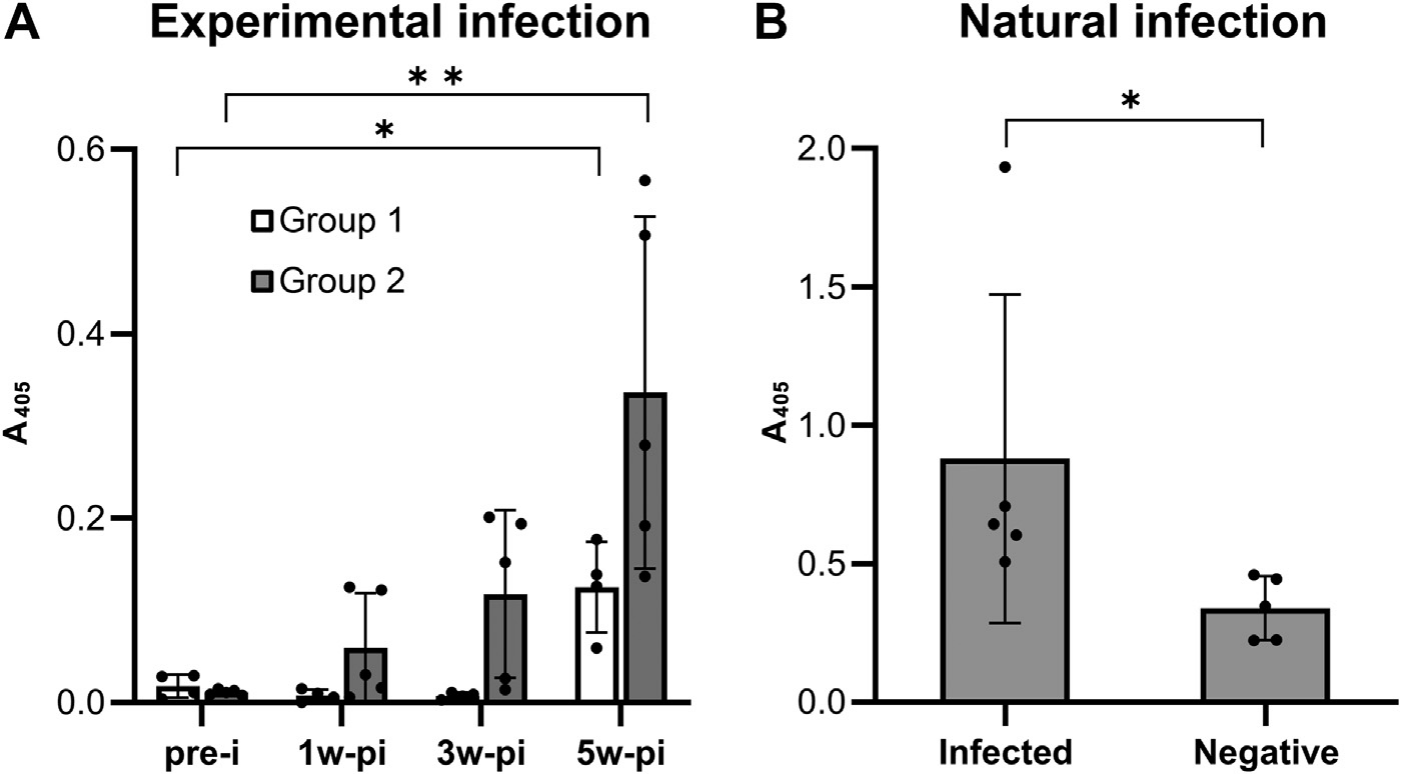
**Recognition of FhCyLS-2 by serum antibodies from *F. hepatica* infected ruminants.** Sera response to recombinant FhCyLS-2 was analyzed by ELISA and expressed as means ± SD (∗*p* < 0.05, ∗∗*p* < 0.01). *A*, sera from two groups of experimentally infected sheep were drawn prior to infection (pre-i) with *F. hepatica* metacercaria and 1, 3, and 5 weeks post inoculation (1 to 5w-pi). Statistical significance compares pre-i and 5w-pi data. *B*, sera from cattle naturally infected with *F. hepatica* collected from a slaughterhouse are compared with sera from noninfected cattle).

Sera from experimentally infected sheep and from naturally infected cows were examined for reactivity with recombinant FhCyLS-2 in an ELISA assay. Experimentally infected sheep recognized FhCyLS-2 3 weeks post infection, and the ELISA signals steadily increased until week 5 post infection, when the experiment had to be terminated (Fig. 7*A*). In addition, sera from naturally infected cattle, in which *F. hepatica* infections were demonstrated by the presence of *F. hepatica* eggs in the bile of slaughtered animals, reacted significantly stronger with FhCyLS-2 than sera from noninfected cattle (Fig. 7*B*).

These results further support the conclusion that FhCyLS-2 is an actively secreted protein from *F. hepatica*. Moreover, secreted FhCyLS-2 is recognized by the host during *F. hepatica* infection, which suggests that FhCyLS-2 may have potential value as a diagnostic marker for fasciolosis.

## Discussion

In this work, we provide a comprehensive structural, functional, and phylogenetic analysis of FhCyLS-2, an unusual member of the cystatin superfamily from *F. hepatica*. The sequence and spatial structure revealed that FhCyLS-2 combines, in an unprecedented way, hallmarks of two major families in the cystatin superfamily, stefins and true cystatins. Homology in the protein structure indicated a close relationship of FhCyLS-2 to stefins; however, FhCyLS-2 also contains the secretion signal peptide and disulfides, two structural features typical of true cystatins. We demonstrated that FhCyLS-2 was present in the E/S products of adult parasites and was released most likely from their gut. The secreted FhCyLS-2 was recognized by the immune system of the host during *F. hepatica* infection, leading to production of specific antibodies detected in the serum. The crystallographic analysis showed that FhCyLS-2 incorporated two disulfide bridges into the disulfide-free scaffold of stefins, and the spatial pattern of disulfides was related to but not identical with that of true cystatins. The acquisition of disulfides might reflect the secretion status of FhCyLS-2 that differs from that of typical intracellular stefins. In general, disulfides stabilize proteins in the oxidizing extracellular environment, and therefore, they are present in the majority of secreted proteins while they are rather rare in cytosolic proteins (34).

**Figure 8:**
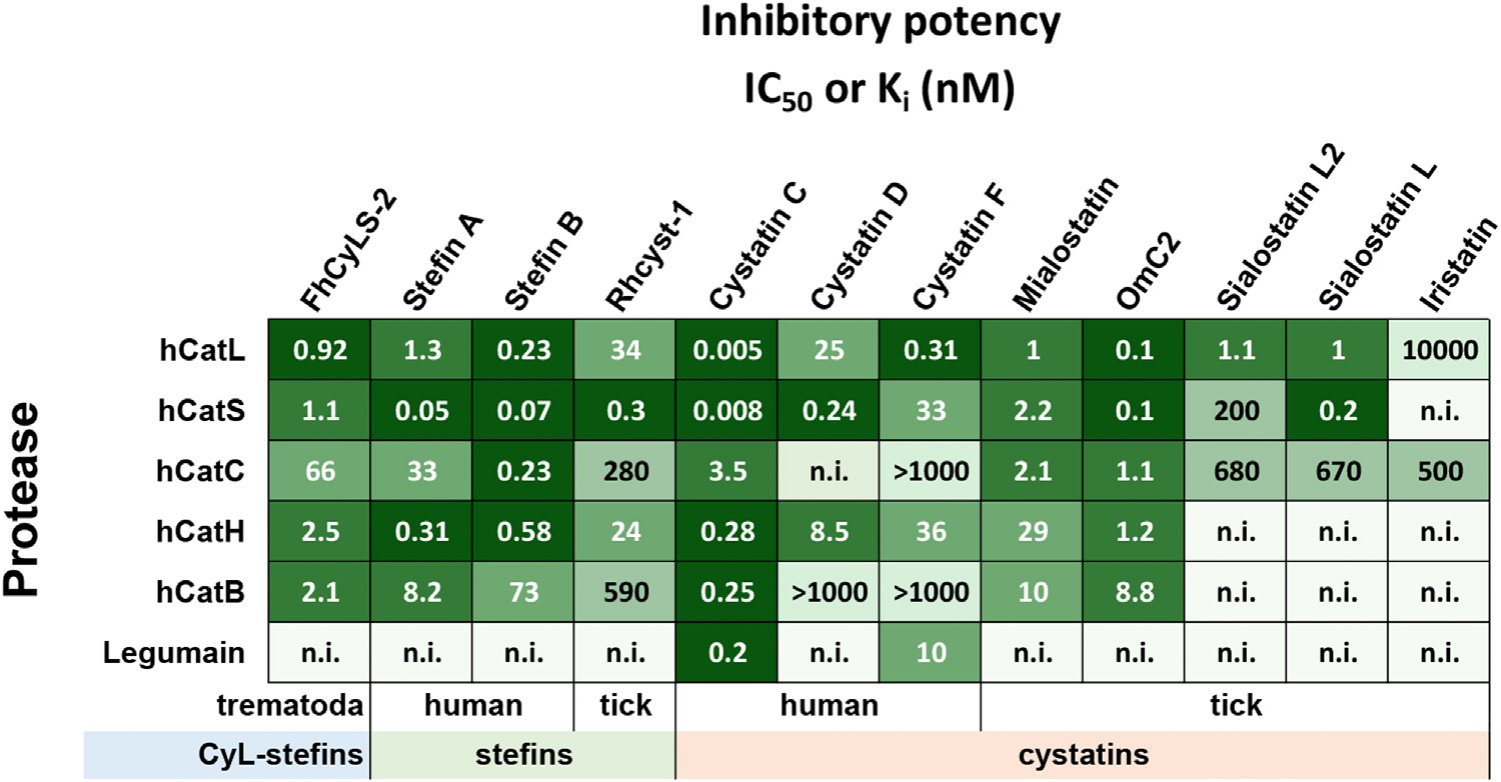
**Comparison of the inhibitory potency of FhCyLS-2 and other members of the cystatin superfamily.** Well-documented classical stefins from the type 1 family and true cystatins from the type 2 family are presented, including members of human origin (stefins A and B, cystatins C, D, and F) and those from parasitic ticks (salivary sialostatins L and L2 and iristatin; salivary gland-associated Rhcyst-1; gut-associated mialostatin and OmC2). Inhibition data against various cysteine proteases, including human papain-family cathepsins L to B (hCatL to hCatB) and mammalian legumains, are presented as IC_50_ (tick inhibitors (10, 37, 69, 70, 71), FhCyLS-2 data are from Table 1) or K_i_ values (human inhibitors (4, 72, 73, 74, 75, 76)) and colored as a heatmap (*green scale*); n.i. means no inhibition.

The FhCyLS-2 molecule bears the reactive center with an overall architecture common to its superfamily and is functionally competent for protease binding. FhCyLS-2 acts as an effective multitarget inhibitor of cysteine cathepsins of mammalian as well as *F. hepatica* origins. Figure 8 compares inhibitory specificity of FhCyLS-2 with that of other members of the superfamily. It shows that FhCyLS-2 effectively inhibits cysteine cathepsins with a wide range of endo- and exopeptidase cleavage specificities and thus resembles several broad-specificity inhibitors, including previously well-characterized human and tick stefins, gut-associated tick cystatins, and also some secreted human cystatins. Mammalian cysteine cathepsins sensitive to FhCyLS-2 play an essential role in a variety of immunological mechanisms such as antigen processing and activation of neutrophils, cytotoxic T lymphocytes, and natural killer cells (for review, see (35, 36)).

Therefore, we hypothesize that secreted FhCyLS-2 mimics specific host-derived cystatins and interferes with their functions *in vivo*, controlling cathepsin-mediated proteolysis as previously found for cystatins with various immunomodulatory activities produced by helminths or ticks (for review, see (11, 16)). Furthermore, FhCyLS-2 inhibits secreted digestive cathepsins L from *F. hepatica* and may function as a physiological regulator in order to protect parasite tissues from autoproteolytic damage. Thus, FhCyLS-2 might have a dual role in the control of both exogenous and endogenous proteolytic systems during host–parasite interactions. Of note is that a similar combined function was proposed for the tick cystatin OmC2, which is secreted into the host for immunomodulatory action as well as regulates proteolysis in the tick gut (37).

The sequence and structural features and phylogenetic analysis revealed that FhCyLS-2 is a member of the type 1 family of stefins. We identified a set of FhCyLS-2 homologues in certain trematode and myxozoan parasite groups. They are evolutionarily different from classical stefins and form a distinct stem group in the phylogenetic branch of the type 1 family. Based on this, we defined a new group called cystatin-like stefins (CyL-stefins) within the cystatin superfamily, with FhCyLS-2 as a prototype member. They possess an obligatory signal peptide on the stefin-like sequence lacking the C-terminal motif Asp/Glu-Xxx-Leu-Xxx-Tyr/His-Phe. The structure of CyL-stefins typically contains two disulfides; however, their number can be reduced to one or, rarely, altogether absent.

A vast majority of animals express both classical stefins and true cystatins as important antiproteolytic molecules (5, 6). We analyzed the repertoire of cystatin superfamily genes, including CyL-stefins, among individual fluke groups and found significant differences when comparing Plagiorchiida, Opisthorchiida, and Schistosomatida. *F. hepatica* and all other liver and intestinal foodborne flukes (Plagiorchiida) encode both CyL-stefins and classical stefins but lack genes for true cystatins. Conversely, no CyL-stefins were identified in the blood flukes (Schistosomatida) that possess classical stefins and true cystatins; however, opisthorchiid flukes combine all three inhibitor groups. In addition, Plagiorchiida and Opisthorchiida also express multicystatins. We hypothesize that CyL-stefins represent an evolutionary upgrade of classical stefins that occurred as a molecular adaptation to compensate for the absence of, or insufficient functional range of, secreted true cystatins. CyL-stefins can form a multigene group (*e.g.*, three homologues present in *F. hepatica*), and this diversification burst, also known in true cystatins, is unusual compared with classical stefins, which remain rather evolutionarily stable without dynamic gene duplications (6). What drives the differences in the repertoire of superfamily genes among fluke taxa? It is tempting to speculate that they reflect the complexity of life cycles of flukes and strategies associated with parasite development and survival in host organs and avoidance of host immunity. In general, a broader panel of superfamily members is associated with a higher complexity of multihost life cycles, the pronounced involvement of vertebrate hosts possessing more advanced immunity than invertebrate hosts, and diverse types of invaded tissues with different spectra of host proteases (38, 39). The overall complexity of these factors decreases from Opisthorchiida to Plagiorchiida and Schistosomatida, in line with their inhibitor repertoire (File S1).

In summary, we present a comprehensive structural and functional characterization of FhCyLS-2, a secreted inhibitor of cysteine cathepsins from *F. hepatica*, and propose its potential role in host–parasite interactions. The unique sequence and structural pattern of FhCyLS-2, together with phylogenetic analysis of its homologues, allowed us to define cystatin-like stefins as a new distinct group belonging to the cystatin superfamily.

## Experimental procedures

### Parasites and parasite-derived materials

Adult worms of *F. hepatica* were collected from the livers of naturally infected cattle acquired from the local abattoir. The worms were washed three times in wash medium (RPMI 1640 medium [Gibco] supplemented with Penicillin-Streptomycin-Amphotericin B [Antibiotic Antimycotic 100×, Gibco] and 10 mM HEPES, pH 7.3) and then transferred to complete culture medium (RPMI 1640 medium supplemented with 2 mM L-glutamine supplemented with 5% calf serum, 55 mM glucose, 30 mM HEPES, penicillin [100 U/ml], streptomycin [100 μg/ml], and gentamicin [25 μg/ml], pH 7.3). After 4-h incubation at 37 °C, the medium with freshly laid eggs was collected. Metacercariae were obtained from Ridgeway Research Ltd. NEJ material was prepared by excystation of metacercariae (19) and harvested after 24 h cultivation. For protein extraction, the worm tissue in PBS containing protease inhibitor cocktail (539131, Calbiochem) was mechanically disrupted by metal beads (Qiagen TissueLyser II), following by ultrasonication. The lysate was centrifuged at 16,000*g* at 4 °C for 10 min, and the supernatant was stored at −80 °C. Medium containing the E/S products of adult flukes was collected after cultivation for 4 h at 37 °C in serum-free medium, filtered through an Ultrafree-MC 0.22 mm filter (Millipore), concentrated, and stored at −80 °C.

### Ethic statement

Animal experiments were carried out at the experimental units of the Vetsuisse Faculty at the University of Zurich after approval by the Cantonal Veterinary Office of Zurich (permission numbers 162/2009 for mice and 234/2012 for sheep) according to Swiss animal rights and regulation standards.

### Isolation of mRNA, cDNA synthesis, and qRT-PCR

Total RNA was extracted from *F. hepatica* developmental stages using Trizol reagent (Invitrogen) and purified using the RNA Isolation Kit RNeasy (Qiagen) as reported (40). Single-stranded cDNA was synthesized from 1 μg of total RNA using SuperScript III reverse transcriptase (Invitrogen) and an oligo d(T)18 reverse primer according to the manufacturer's protocol, and the final cDNA product was purified (QIAquick PCR Purification Kit, Qiagen). The gene expression profile of FhCyLS-2 was assessed using qRT-PCR. The primers (two sets of forward and reverse primers, Table S4) were designed to amplify 160 to 180 bp fragments using the Primer 3 software (http://frodo.wi.mit.edu/cgi-bin/primer3/primer3_www.cgi), and their efficiency was evaluated as described (40, 41). Triplicate reactions, containing SYBR-green MasterMix (Roche), were carried out in a final volume of 25 μl in 96-well plates in an MX 3005P Real-Time PCR cycler (Bio-Rad). The amplification profile consisted of an initial hot start (95 °C for 10 min) followed by 40 cycles comprising 95 °C for 30 s, 55 °C for 60 s, and 72 °C for 30 s. Carboxyrhodamine and *F. hepatica* cytochrome C oxidase 9, subunit I (GenBank: KF111595.1) were used as a reference dye and gene, respectively. Upon completion of the amplification, the dissociation curve was examined for potential primer dimerization. The cycle threshold values were averaged, and the standard deviation was determined. The relative expression levels were calculated using the delta–delta cycle threshold method (42). The PCR reactions were performed in duplicate for each cDNA sample obtained from a pool of multiple parasites of individual developmental stages. Statistical significance was determined with the unpaired Student's *t* test.

### Cloning and expression of recombinant FhCyLS-2

FhCyLS-2 (GenBank: AY647146) was expressed in the X-33 strain of the methylotrophic yeast *P. pastoris* (Thermo Fisher). The full-length FhCyLS-2 gene without the N-terminal signal sequence predicted by SignalP v5.0 (43) was amplified from the adult stage cDNA using the forward primer 5′-CGGAATTCGAGGTGAAATGCTCGTGGGTG-3’ (EcoRI restriction site underlined) and reverse primer 5′-CGTCTAGAGCTCA**ATGATGATGATGATGATG**AGCTGCAGCAGTGCAGGATACCCGAGTC (XbaI restriction site underlined, 6xHis-tag in bold) with Phusion DNA polymerase (Fermentas). The insert sequence was cloned into pPICZαB vector (Thermo Fisher) using EcoRI and XbaI restriction sites. Recombinant FhCyLS-2 contained an N-terminal extension of residues AGIRG and a C-terminal extension of residues AAAHHHHHH compared with the native mature sequence. The vector sequence was verified by DNA sequencing. Transformation of *P. pastoris* cells and protein expression were carried out as described (44). Media supernatants from induced *P. pastoris* were lyophilized and stored at −20 °C until use.

### Purification of recombinant FhCyLS-2

The recombinant His-tagged FhCyLS-2 was purified from the concentrated and desalted expression medium by Ni-affinity chromatography on a HiTrap IMAC HP column (GE Healthcare) using the manufacturer’s protocol for elution with imidazole at pH 8. It was followed by size-exclusion chromatography, using a HiLoad 16/600 Superdex 75 pg column (GE Healthcare) equilibrated with 50 mM Tris-HCl, 200 mM NaCl, pH 8. The purified protein was concentrated and buffer-exchanged into 10 mM Tris-HCl, 10 mM NaCl, pH 8, using an Amicon Ultra-3K centrifugal unit (Millipore). Purification was monitored by Laemmli SDS-PAGE on 15% polyacrylamide gels stained by Coomassie Brilliant Blue R-250. Edman sequencing of purified recombinant FhCyLS-2 demonstrated the N-terminal sequence starting with IRG, which indicated a proteolytic trimming and removal of the N-terminal dipeptide AG during protein expression.

### Protein crystallization, data collection, and structure determination

Crystals were grown using vapor-diffusion setup in hanging drops at 18 °C. The ratio of protein to reservoir solution in the drops was 1:1. The drops were equilibrated over 300 μl of reservoir solution consisting of 2 M ammonium sulfate, 0.1 M HEPES, 10% PEG 400, pH 7.5. The protein concentration of the FhCyLS-2 solution was 17 mg/ml. Crystals shaped as thin needles were soaked overnight in 2 M ammonium sulfate, 0.1 M Na iodide, 0.1 M HEPES, 10% PEG 400, pH 7.5 and flash cooled in liquid nitrogen. Diffraction data were collected at 100 K on the MX14.1 beamline at the BESSY electron-storage ring, Berlin, Germany (45). The anomalous dataset was collected at a wavelength of 1.500 Å; the native dataset was collected at a wavelength of 0.9184 Å. Diffraction data were integrated, reduced, and scaled using the X-ray Detector Software (46). Phasing was performed according to the SIRAS method (47), using anomalous and native datasets with help of hkl2map (48) and SHELX (49). The initial model was improved and rebuilt by Bucaneer (50), refined by REFMAC5 (51), manually rebuild in Coot (52), and validated with MolProbity (53), all programs originating from the CCP4 package (54). The structure was refined at 1.60 Å resolution, with R_work_ = 0.22 and R_free_ = 0.26. The effective resolution d_eff_ of the structure calculated using d_eff_ = d_min_C^−1/3^ (55) was 1.61 Å, and the resolution at the mean I/sigma(I) = 2 was 1.74 Å. Figures showing structural representations were generated by PyMOL (56). Table S3 was generated by PHENIX (57). The atomic coordinates and experimental structure factors were deposited in the Protein Data Bank with accession code 6I1M.

### Protease inhibition assays

Inhibition measurements were performed in triplicate in 96-well microplates (100 μl assay volume) at 37 °C. Recombinant FhCyLS-2 was preincubated with protease for 15 min followed by the addition of a specific fluorogenic substrate (see below). The kinetics of product release were continuously monitored using an Infinite M1000 (Tecan) microplate reader at 365 nm excitation and 450 nm emission wavelengths (for AMC-containing substrates) or at 330 nm excitation and 410 nm emission wavelengths (for Abz-containing substrate). IC_50_ values were determined from residual velocities using dose–response plots; nonlinear regression was fitted using GraFit software (Erithacus). All substrates were purchased from Bachem with the exception of Abz-Phe-Arg-Phe(NO_2_)-OH from MP Biomedicals. Human cathepsins L, K, and V were purchased from Merck; cathepsins F, B, and S from Enzo Life Science; and cathepsin X and legumain from R&D Systems*. Carica papaya* papain was from Merck, bovine cathepsins C and H were prepared as described in (58, 59), and *F. hepatica* cathepsins L1, L2, and L3 were prepared as described in (60, 61). Substrates and proteases were applied in assays as follows: *Z*-Phe-Arg-AMC in 2.5 μM concentration with 0.2 nM cathepsin L, 4.3 μM with 0.2 nM cathepsin K, 3.6 μM with 0.1 nM cathepsin V, 53 μM with 0.2 nM cathepsin B, 27 μM with 1.2 nM cathepsin F, 100 μM with 0.5 nM papain, 20 μM with 2 nM FhCL1, and 20 μM with 5 nM FhCL2; 17 μM *Z*-Val-Val-Arg-AMC with 0.1 nM cathepsin S; 100 μM H-Arg-AMC with 1.9 nM cathepsin H; 87 μM Abz-Phe-Arg-Phe(NO_2_)-OH with 1.2 nM cathepsin X; 130 μM H-Gly-Arg-AMC with 0.4 nM cathepsin C; 70 μM *Z*-Ala-Ala-Asn-AMC with 1.2 nM legumain; and 20 μM Z-Gly-Pro-Arg-AMC with 6 nM FhL3. The assay buffers were as follows: 100 mM Na acetate, pH 5.0 (for cathepsin X, legumain, and E/S products) or pH 5.5 (for cathepsins L, K, V, B, F, C, and papain); 50 mM MES, pH 5.5 (FhCL1 and FhCL3) or pH 6.0 (FhCL2) or pH 6.5 (for cathepsins S and H); the buffers contained 2.5 mM dithiothreitol, 0.1% polyethylene glycol 1500, and 50 mM NaCl (for cathepsin C).

### Immune assays

Production of anti-FhCyLS-2 serum: Polyclonal serum was raised in three 12-week-old NMRI mice initially immunized with 60 μg recombinant FhCyLS-2 in Freund complete adjuvant (100 μl s.c.) followed by a single booster injection with the same amount of antigen in Freund incomplete adjuvant after 3 weeks. The collected serum was tested in ELISA for immunoreactivity against recombinant FhCyLS-2, as described for sheep serum below, with the only difference being the use of mouse serum (1:200) and anti-mouse-IgG for detection (1:10,000, A3562, Sigma) and in Western blot for specificity against the *F. hepatica* E/S products and total worm extract containing native FhCyLS-2.

Immunofluorescence microscopy: Adult *F. hepatica* worms were prepared for immunofluorescence microscopy as described (62, 63). Antibody dilutions were 1:500 for mouse polyclonal anti-FhCyLS-2 serum and 1:200 for anti-mouse IgG Alexa 633–conjugated secondary antibody (A21052, Molecular Probes). Antibodies were incubated for 45 min at 25 °C on sections with 3 washes between the primary and secondary antibody and 4 times after secondary antibody; the third wash contained 1 μg/ml DAPI. The sections embedded in Mowiol were visualized using a Leica SP2 AOBS confocal laser scanning microscope (Leica Microsystems) using a 20× oil immersion objective, and images were processed using the Huygens deconvolution software package version 2.7 (Scientific Volume Imaging).

Immunoreactivity of sera from infected animals: Sheep were assigned to groups 1 and 2, consisting of 4 and 5 animals, respectively, that were experimentally infected with 150 metacercariae in a gelatin capsule administered orally. Prior to infection, group 2 was immunized with ovalbumin (300 μg) as an additional and irrelevant immunogen three times at two-weekly intervals. The infection was allowed to proceed for 5 weeks, and sera were collected. Sera from cattle (10 animals) were collected at abattoir, and bile was taken from gall bladders and examined for the presence of *F. hepatica* eggs (64).

ELISA assay: Microtiter plates were coated with recombinant FhCyLS-2 (0.5 μg per well), blocked with PBS containing 2% dry milk and 0.05% Tween-20. After three washes with PBS containing 0.05% Tween-20 (PBS-T), the antigen was incubated with sera (1:200 sheep, 1:100 cattle) for 1 h at 22 °C. Unbound antibody was removed by washing 4 times with PBS-T, and the plate was incubated with alkaline phosphatase–conjugated secondary antibodies (donkey anti-sheep IgG 1:10,000, A24564, Invitrogen or goat anti-bovine IgG 1:2000, 5220-0383, KPL-SeraCare) for 1 h at 22 °C. The plate was washed 4 times with PBS-T and incubated with the substrate (pNPP disodium hexahydrate, Sigma) for 15 min and read at 405 nm (with reference at 630 nm). All measurements were performed in duplicate. Statistical significance was determined with the two-sided Mann–Whitney *U* test.

Western blot: For testing mice sera, protein samples (10 μg of *F. hepatica* E/S products and adult worm lysate, or 1 μg of FheCyLS-2) were separated by reducing Laemmli SDS-PAGE and electroblotted onto a nitrocellulose membrane. The membrane was blocked with PBS-T, containing 5% dry milk, and incubated with polyclonal serum (1:200) in blocking solution for 4 h at 22 °C. After six washes with PBS-T, bound antibodies were detected with horseradish peroxidase–conjugated secondary antibodies (goat anti-mouse IgG, 1:20,000, 1706516, Bio-Rad) for 2 h at 22 °C. Signal was visualized by Western Lightning Chemiluminescence Reagent (PerkinElmer Life Sciences).

### Mass spectrometry proteomic analysis

For identification of native FhCyLS-2, proteins of the E/S products from adult *F. hepatica* were reduced, alkylated, and digested with trypsin and chymotrypsin. Recombinant FhCyLS-2 was analyzed analogously using trypsin digest. The LC-MS/MS analysis of the digests was performed on an UltiMate 3000 RSLCnano system (Dionex) coupled to a TripleTOF 5600 mass spectrometer with a NanoSpray III source (AB Sciex). The peptides were separated on an Acclaim PepMap100 analytical column (3 μm, 15 cm × 75 μm; Thermo Scientific) with gradient elution in a 0.1% formic acid/acetonitrile system. Full mass spectrometry scans were recorded from 350 to 1250 *m/z*, up to 25 candidate ions per cycle were subjected to fragmentation; in MS/MS mode the fragmentation spectra were acquired from 100 to 1600 *m/z*. The mass data were processed by the ProteinPilot 4.5 software (AB Sciex).

For disulfide pairing analysis, the recombinant FhCyLS-2 was digested with trypsin and subsequently with GluC. The LC-MS/MS analysis of the digests was performed using the same setup as described above. A peptide of molecular weight 3769.656 in the form of multiply charged (6+) ion (629.286) was detected, and its MS/MS spectrum was recorded (Table S1). This peptide matches the sequence of a cluster of three peptides (LLTAGSVVSSC^39^E, VSGGATC^66^PGC^69^WE, VSC^95^TAAAHHHHHH) connected by two disulfide bonds (Table S1). The MS/MS fragments with *m/z* 1016.932 (doubly charged) and 678.291 (triply charged) demonstrated the breakage of the parental cluster forming a cluster of two peptides (PGC^69^WE, VSC^95^TAAAHHHHHH) connected by the disulfide Cys69-Cys95. This indicated that the disulfide connectivity in the original peptide cluster was provided by disulfide bonds of Cys69-Cys95 and Cys39-Cys66.

### Phylogenetic and bioinformatic analyses

The dataset used for the phylogenetic analysis consisted of 160 amino acid sequences of vertebrate and invertebrate single-domain cystatins. It includes the recently reported dataset of the superfamily members (30) and newly mined helminth homologues. Sequences were aligned in MAFFT version 7.450 (65) implemented in Geneious Prime version 2019.0.4 (66) using the G-INS-i method, with default settings for gap opening penalty and offset value. The sequences were retrieved as GenBank/WormBase Parasite annotated entries or were mined from the publicly available genome/transcriptome assemblies using the tBLASTn algorithm and E-value cutoff 10^−5^ (File S1). Multidomain cystatins (listed for helminths in File S1) were not included in the analysis due to difficulties with their alignment to single-domain molecules and classification of their secretion status. Nonhomologous terminal regions of the alignment were removed manually in Geneious Prime so the final alignment comprised 164 positions, principally corresponding to the conserved cystatin domain Pfam PF00031. The metamonad *Giardia intestinalis* cystatin (GenBank: XP_001705664) resembling the most ancestral eukaryotic cystatin (6) was used as the outgroup. The phylogenetic tree was reconstructed by the maximum likelihood method in IQ-TREE version 1.6.12 (67) using the WAG+F+G4 protein model selected by ModelFinder (68). Nodal supports were based on 1000 bootstrap replicates. The visualization and graphics of the resulting tree was performed with the FigTree version 1.4.2. The presence/absence of signal peptide in the cystatin sequences was predicted in SignalP version 5.0 (43).

## Data availability

The 3D structure of FhCyLS-2 obtained by X-ray crystallography was deposited in the PDB database with the accession code 6I1M.

## Supporting information

This article contains supporting information.

## Conflict of interest

The authors declare that they have no conflicts of interest with the contents of this article.
