# Supplementary material for: An evolutionary molecular adaptation of an unusual stefin from the liver fluke Fasciola hepatica redefines the cystatin superfamily
Source: J Biol Chem. 2023 Feb 1;299(3):102970. doi: 10.1016/j.jbc.2023.102970 (PMC9986714; doi:10.1016/j.jbc.2023.102970)
Supplement: Supplemental data [file mmc1.pdf]

An evolutionary molecular adaptation of an unusual stefin from the liver fluke *Fasciola hepatica* redefines the cystatin superfamily

Michal Buša, Zuzana Matoušková, Pavla Bartošová-Sojková, Petr Pachl, Pavlína Řezáčová, Ramon Marc Eichenberger, Peter Deplazes, Martin Horn, Saša Štefanić, and Michael Mareš

Supporting information

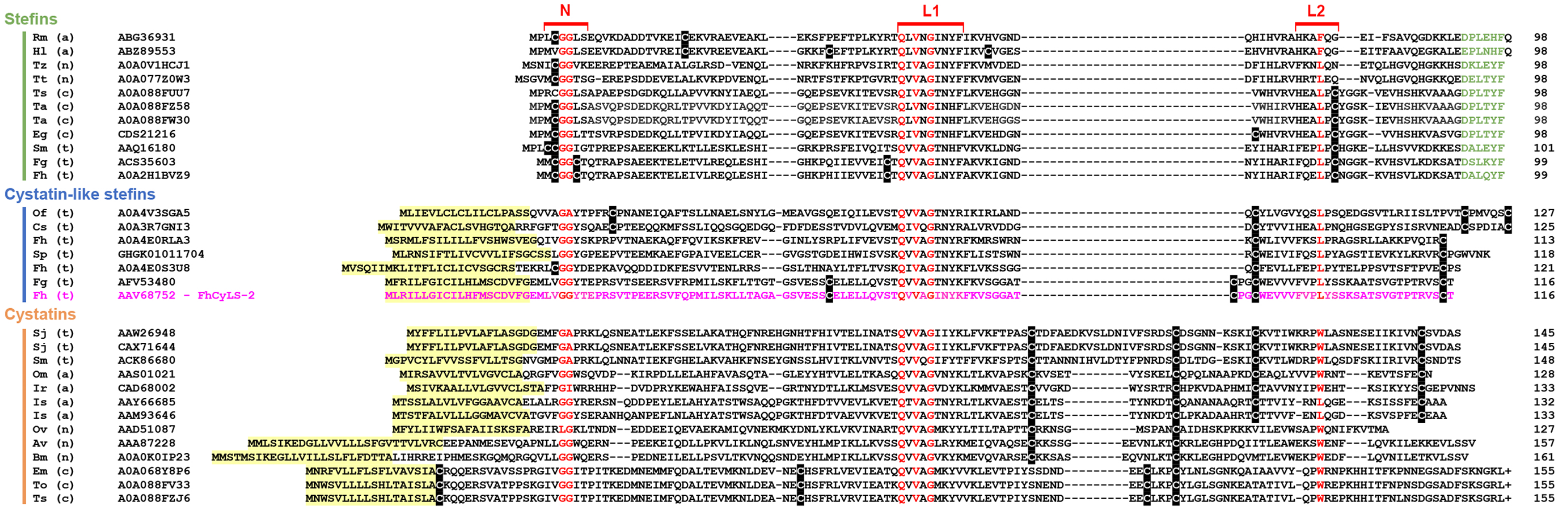

Figure S1. Multiple sequence alignment of FhCyLS-2 with selected members of the cystatin superfamily from blood feeding parasites. The groups of sequences correspond to classical stefins from the type 1 family, true cystatins from the type 2 family, and cystatin-like stefins. The sequence of FhCyLS-2 is in magenta. The predicted signal peptides are highlighted in yellow, and cysteine residues in mature protein sequences are shaded black. The conserved C-terminal segment of classical stefins is green. Three segments N, L1, and L2 forming the reactive center for inhibition of cysteine cathepsins are indicated by red bars and labeled; the critical consensus positions are shown in red. The sequence length is indicated on the right (additional C-terminal extensions are marked with a cross). The sequences are indicated with an abbreviated species name (Av: *Acanthocheilonema viteae*, Bm: *Brugia malayi*, Cs: *Clonorchis sinensis*, Em: *Echinococcus multilocularis*, Eg: *Echinococcus granulosus*, Fg: *Fasciola gigantica*, Fh: *Fasciola hepatica*, Hl: *Haemaphysalis longicornis*, Ir: *Ixodes ricinus*, Is: *Ixodes scapularis*, Ov: *Onchocerca volvulus*, Of: *Opisthorchis felineus*, Om: *Ornithodoros moubata*, Rm: *Rhipicephalus microplus*, Sj: *Schistosoma japonicum*, Sm: *Schistoma mansoni*, Sp: *Sphaeridiotrema pseudoglobulus*, Ta: *Taenia asiatica*, Ts: *Taenia saginata*, To: *Taenia solium*, Tt: *Trichuris trichiura*, Tz: *Trichinella zimbabwensis*), phylogenetic affiliation in parentheses (a: arthropoda, n: nematoda, c: cestoda, t: trematoda), and GenBank accession number.

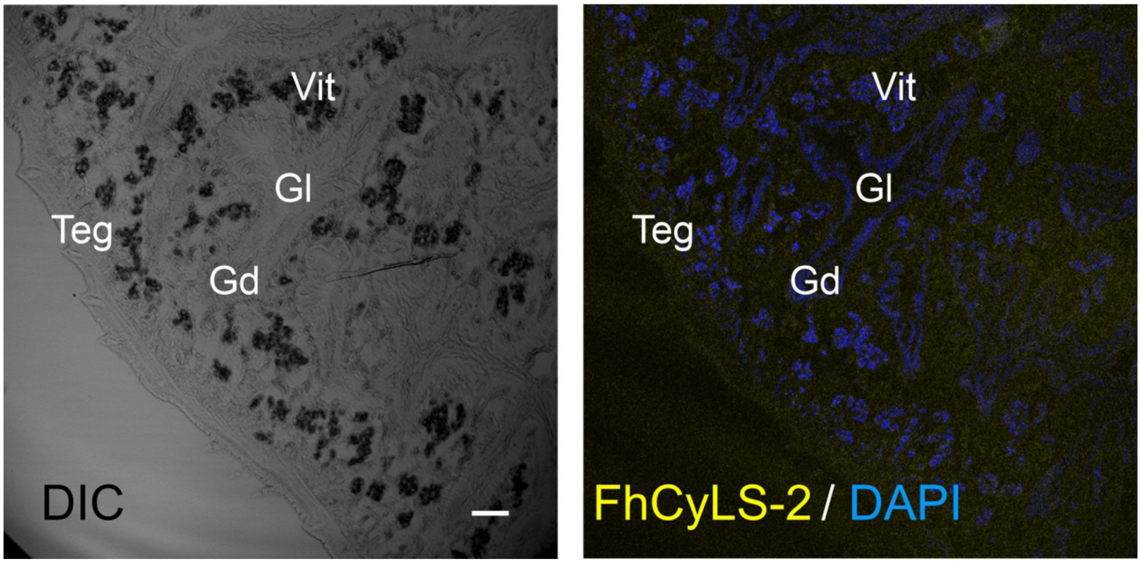

Figure S2. Negative control staining of section of adult *F. hepatica*. Semi-thin tissue section of the adult worm was probed with pre-immune mice serum followed by reaction with an anti-mouse IgG Alexa 633-labeled secondary antibody (yellow). DAPI was used to label nuclear DNA (blue). The left panel shows differential interference contrast (DIC), the right panel fluorescent channels. Positive staining with antiserum is presented in Figure 3. Gd: gastodermal cells, Gl: gut lumen, Teg: tegument, Vit: vitelline cells; scale bar represents 100 μm.

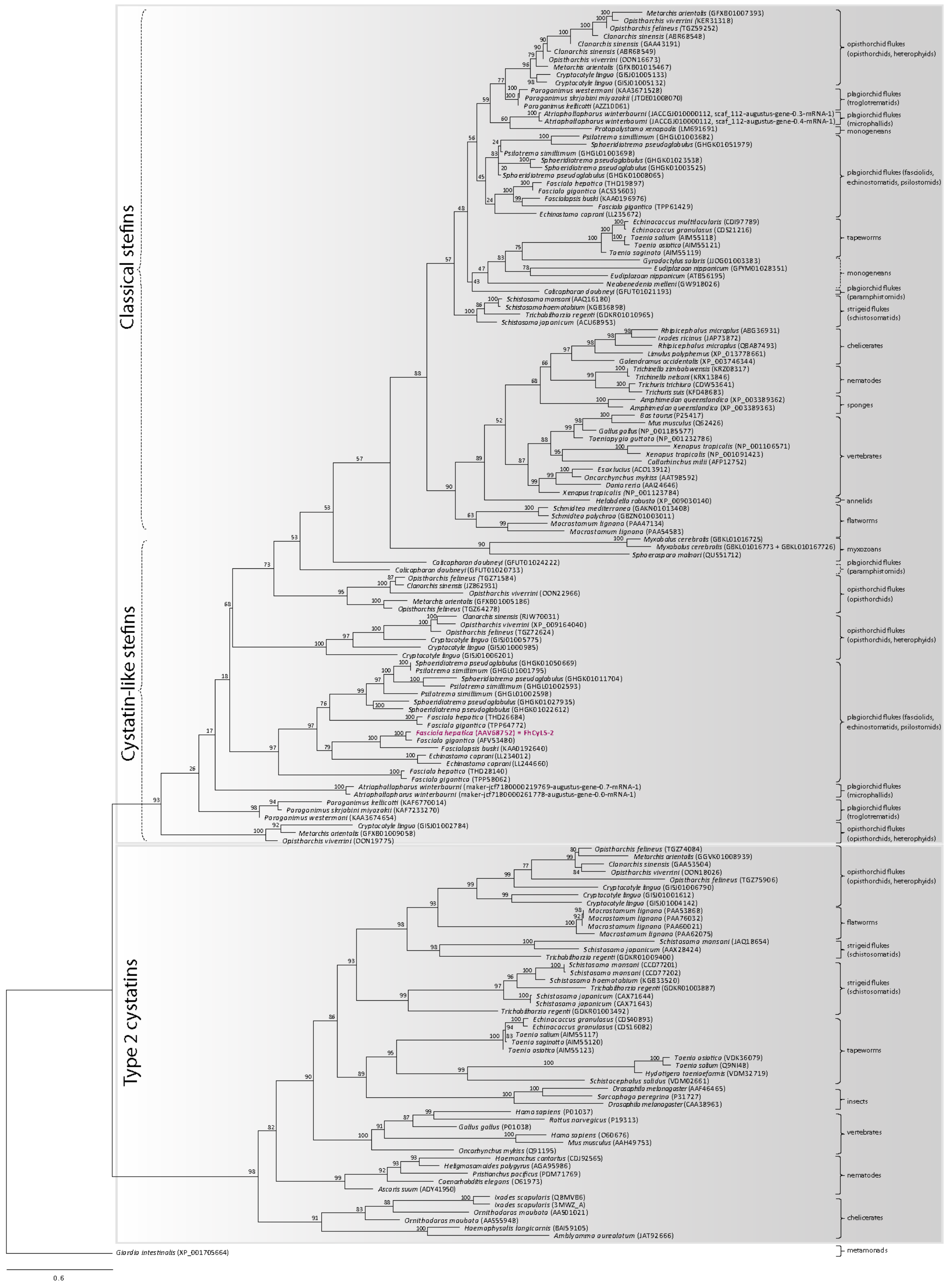

Figure S3. Maximum likelihood phylogenetic tree of the cystatin superfamily. The rooted, non-collapsed tree of vertebrate and invertebrate members shows two clades of the type 1 family of stefins and the type 2 family of true cystatins; the former is divided into classical stefins and cystatin-like stefins. The highest taxonomic rank is indicated. FhCyLS-2 is in magenta. The *Giardia intestinalis* cystatin was used as the outgroup. Nodal supports calculated from 1000 bootstrap replicates are indicated.

Table S1. Mass spectrometry analysis of posttranslational modifications of FhCyLS-2. Listed are selected peptides derived from the native and recombinant FhCyLS-2 that cover the mature N-terminal region (tryptic fragment 1–11 and chymotryptic fragment 8–21) and the regions containing disulfide-bridged cysteine residues. The peptides were analyzed by LC-MS/MS as described in Experimental procedures. Cysteine residues (red) forming a disulfide bond in the peptide cluster are aligned; the disulfide bonds connect residues Cys39 to Cys66 and Cys69 to Cys95. Delta indicates the difference between experimental and theoretical mass.

FhCyLS-2	Theoretical peptide mass [M] (Da)	Measured peptide mass [M] (Da)	Delta (Da)	Peptide sequence (residues No.)
Native	1250.597	1250.594	0.002	**EMLVGGYTEPR** (1-11)
Native	1632.811	1632.815	0.005	**TEPRSVTPEERSVF** (8-21)
Recombinant	3769.656	3769.671	0.015	**LLTAGSVVSSCE** (29-40)<br>**VSGGATCPGCWE** (59-71)<br>**VSCTAAAHHHHHH** (93-105)

Table S2. Inhibitory effect of FhCyLS-2 on the proteolytic activity of the E/S products. The proteolytic activity of the E/S products of adult *F. hepatica* was assayed using the fluorogenic peptide substrate Z-Phe-Arg-AMC and three different chromogenic protein substrates. The inhibition by 0.5 μM FhCyLS-2 is expressed as remaining activity relative to the uninhibited control (100%) and is compared with the effect of 0.5 μM E-64, a general peptide inhibitor of papain-family proteases. The mean values ± SE are given.

Substrate	Relative inhibition (%)	
	**E-64**	**FhCyLS-2**
Z-Phe-Arg-AMC	99.7 ± 0.8	99.1 ± 0.4
Azocasein	99.2 ± 0.7	96.3 ± 1.9
Azocollagen	99.4 ± 4.1	93.9 ± 0.8
Elastin congo red	98.4 ± 1.2	98.2 ± 3.0

Table S3. Crystal parameters, data collection statistics, and refinement statistics. All statistics were generated by the program PHENIX (see Experimental procedures).

**Dataset**	**Native**	**I^- soaked**
Wavelength (Å)	0.918	1.5
Resolution range (Å)	46.67 – 1.60 (1.66 – 1.60)	33.6 – 1.90 (1.97 – 1.90)
Space group	$C222_1$	$C222_1$
a, b, c (Å)	64.9, 67.2, 44.5	64.7, 67.2, 44.6
α, β, γ (°)	90.0, 90.0, 90.0	90.0, 90.0, 90.0
Total reflections	88303 (8518)	89387 (5006)
Unique reflections	13131 (1297)	7925 (727)
Multiplicity	6.7 (6.6)	11.3 (6.5)
Completeness (%)	98.43 (99.69)	97.97 (91.50)
Mean I/sigma(I)	7.83 (0.80)	17.81 (2.77)
Wilson B-factor (Å^2)	21.33	22.25
R_{merge}	0.16 (2.14)	0.11 (0.63)
R_{meas}	0.17 (2.32)	0.12 (0.69)
R_{pim}	0.07 (0.90)	0.03 (0.27)
$CC_{1/2}$	1.00 (0.43)	1.00 (0.91)
CC^*	1.00 (0.77)	1.00 (0.98)
Reflections used in refinement	12986 (1297)	
Reflections used for R_{free}	650 (65)	
R_{work}	0.22 (0.44)	
R_{free}	0.26 (0.36)	
CC_{work}	0.95 (0.41)	
CC_{free}	0.94 (0.24)	
Number of non-hydrogen atoms	807	
macromolecules	707	
ligands	2	
solvent	98	
protein residues	93	
RMS bond length (Å)	0.014	
RMS angle (°)	1.82	
Ramachandran favored (%)	97.8	
Ramachandran allowed (%)	1.1	
Ramachandran outliers (%)	1.1	
Rotamer outliers (%)	1.18	
Clashscore	3.5	
Average B-factor (Å^2)	28.47	
macromolecules (Å^2)	27.44	
ligands (Å^2)	25.70	
solvent (Å^2)	35.93	

Table S4. Primer sets used for qRT-PCR analysis.

Primer name	sequence 5'-3'
QFheCyLS2-A-s	CCCAGTGAGGTTGGATGACT
QFheCyLS-2-A-as	CAGTCTGCAATGACAGCACA
QFheCyLS-2-B-s	TGTTGCGCATACTACTTGGAA
QFheCyLS-2-B-as	AGCAACTCCAACTCACACGA
FheCoxl-A-s	TGGCCTGTTGTGACTGGTTA
FheCoxl-A-as	ACTACCTGCCGAGACAAGAG
FheCoxl-B-s	GGTAGTGTTGTTTGGGCTCA
FheCoxl-B-as	CCACACAACAGGATCCCATA
